# Unusual Distension of the Urinary Bladder

**Published:** 1885-12

**Authors:** F. A. Butterfield

**Affiliations:** Jewell City, Kansas


### DOMESTIC CORRESPONDENCE.

JEWELL CITY, KANSAS, NOV. 3rd, 1885.

TO THE EDITORS OF THE *Chicago Medical Journal and Examiner.*

*Gentlemen:*

The following is an incomplete history of a case which, if deemed interesting, please use as you see proper.

On January 22nd, 1885, I was called to see Mrs. N. J., age twenty-six years, mother of five children, who had been confined to her bed for the past twelve weeks by what her physician called typhoid fever. Her youngest child was about two weeks old when the fever came on. I found her greatly emaciated, delirious, and with tongue heavily coated. Temperature 102° F. Her pulse 128° per minute. There was complete anorexia and some thirst. I was told that she had been "troubled with bloating for about four weeks." Upon inquiry, as to the action of her kidneys, I was told that she passed urine frequently, but only a little at each time, and that she had been taking a diuretic for some time to correct that trouble. Upon passing my hand over the emaciated walls of the abdomen, I could feel the urinary bladder greatly distended. Objection was made to my passing a catheter, as "she passed water nearly all the time." After emptying the bladder, the quantity of urine obtained was so large that I measured it, accurately, and found it to be sixty-one and one-half fluid ounces, or a little more than ten [?] pints. After this the bladder was emptied three or four times during each twenty-four hours. The patient became rational; her tongue cleared off; her appetite improved, and her sleep became natural; but the patient being emaciated more than anything that I ever expected to see, died on the 26th from inanition.

The only point of interest in this case is the great quantity of urine obtained from this patient at one time, which far exceeds the quantity that the bladder is generally believed to be capable of holding. It is a much larger quantity than I have ever heard of, and I thought it might be interesting to others.

Respectfully yours,

F. A. BUTTERFIELD.

